# Molecular Characterization of Extended Spectrum β-lactamase and Carbapenemase Producing *Klebsiella pneumoniae* from a Tertiary Care Hospital

**DOI:** 10.5005/jp-journals-10071-23118

**Published:** 2019-02

**Authors:** Beena Hosdurg Bhaskar, Shalini Shenoy Mulki, Sangeetha Joshi, Ranjeeta Adhikary, Bhavana Malavalli Venkatesh

**Affiliations:** 1,3,4,5 Department of Microbiology, Manipal Hospital, Bengaluru, Karnataka, India; 2 Department of Microbiology, Kasturba Medical College, Mangaluru, Karnataka, India

**Keywords:** AmpC, Carbapenemase, Extended spectrum β-lactamase, Metallo β-lactamase

## Abstract

**Objective:**

The extended-spectrum beta-lactamase (ESBL) and carbapenemase producing gram-negative bacteria among the members of Enterobacteriaceae are of major health concern globally. The present study was carried out to determine proportion and genetic characterization of ESBL and carbapenemase producing *Klebsiella pneumoniae* strains isolated from intensive care units of a tertiary care hospital.

**Materials and methods:**

A total of 250 non-duplicate *K. pneumoniae* isolates were recovered from various clinical specimens from our intensive care units from May 2014 to May 2015. Antibiotic susceptibility testing was performed as recommended by Clinical and Laboratory Standard Institute. Phenotypic identification of ESBL and carbapenemase producing isolates were confirmed by the double-disk synergy test, modified Hodge test, imipenem and imipenem-EDTA combined test, respectively. Molecular characterization of β-lactamase genes were performed by polymerase chain reaction.

**Results:**

Out of 250 *Klebsiella pneumonaie*, 84% isolates were ESBL producers, 66% were carbapenem resistant based on their reduced susceptibility to imipenem, meropenem and ertapenem. Among these 165 carbapenem resistant isolates, 9.7% were positive for *bla*_NDM-1_ and these isolates were also found to be positive for one or more *bla* genes. Co-carriage of AmpC in ESBL and carbapenem resistant isolates were 7.8% and 3.6%, respectively and were negative for *bla*_KPC_ genes.

**Conclusion:**

The study indicated the prevalence of ESBLs and *bla*_NDM-1_, with additional *bla* genes and AmpC among the *K. pneumoniae* isolates in our intensive care units. NDM-1 producing Enterobacteriaceae is a growing health care problem. Detection of the prevalence of antibacterial resistance pattern helps towards improved antibiotic policy and empirical antibiotic treatment.

**How to cite this article:**

Beena HB, Shenoy SM, *et al.* Molecular Characterization of Extended Spectrum β-lactamase and Carbapenemase Producing *Klebsiella pneumoniae* from a Tertiary Care Hospital. Indian J of Crit Care Med 2019;23(2):61-66.

## INTRODUCTION

**I**nfections due to multidrug resistant (MDR) Enterobacteriaceae are an important cause of morbidity and mortality worldwide. Carbapenems are most often used in this treatment. The emergence of resistance to these agents has become a serious health concern globally^1^.

*Klebsiella pneumoniae* is one of the most common Gram- negative bacteria showing resistance to multiple antibiotics. The development of extended-spectrum cephalosporins in the early 1980s was regarded as a major addition to our therapeutic armamentarium in the fight against beta-lactamase mediated bacterial resistance. The emergence of enzymes that have the ability to hydrolyze this cephalosporin's seriously compromised the efficacy of these lifesaving antibiotics. These enzymes were called extended spectrum beta lactamases^2^. Extended spectrum beta-lactamases are plasmid-mediated enzymes that are capable of conferring bacterial resistance to the penicillins, first, second third, fourth generation cephalosporins and aztreonam.

They do this by hydrolysis of these antibiotics but they are inhibited *in vitro* by beta- lactamase inhibitors^3^.

ESBL is predominantly found in *Klebsiella* spp. and*Escherichia coli*, and other members of the Enterobacteriaceae^4^. The most prevalent ESBLs are included in three groups: TEM, SHV and CTX-M^5^. CTX-M type ESBLs show only 40% identity to TEM or SHV ESBLs, but they are closely related to β-lactamases of the *Kluyvera* spp^6^.

Carbapenems were the drug of choice for the treatment of multidrug resistant gram-negative bacterial infections. Emergence of carbapenem resistant bacteria left limited options in the choice of antibiotics to treat the infections caused by them^7^. These bacteria have the potential to spread rapidly within the hospital environment and also across the continents^8^. Resistance to carbapenem is mostly due to production of enzymes-carbapenemases that hydrolyze carbapenems and other β-lactams. Acquired carbapenemases belong to group A (IMI, NMC, SME GES, and *Klebsiella pneumoniae* carbapenemase (KPC), group B metallo-β-lactamase (MBLs of VIM, IMP, GIM, NDM, SIM, and DIM series), and group D (carbapenem hydrolyzing oxacillinases e.g. OXA 48^9^.

NDM-1 producing bacteria are important because the gene encoding this enzyme is located on a transmissible plasmid (of varying size). It is also associated with other resistant determinants leading to extensive drug resistance which is usually exhibited by a majority of the NDM-1 producing enterobacteriaceae leaving only a few therapeutic options.

Therefore NDM-1 producing organisms are also named as “Super bugs”. NDM-1 was first identified and reported in 2009 in *Klebsiellae pneumoniae* and *Escherichia coli*. It was isolated from Swedish patient of Indian origin who was previously hospitalized in New Delhi, India^10^. Indian investigators identified *E. coli* and *Klebsiella* species containing the gene for NDM-1 in multiple geographic regions in India, Pakistan and Bangladesh^11^.

The objective of this study was molecular characterization of the enzymatic mechanisms of resistance to β-lactam antibiotics in *K. pneumoniae*. A multiplex polymerase chain reaction (PCR) was setup for the detection of CTX-M, TEM and SHV genes. The reduced susceptibility to carbapenems by disk diffusion test prompted us to determine the molecular assay on these isolates to detect KPC and NDM-1 genes and also to analyze coexistence of AmpC producers among *Klebsiella pneumoniae* isolates at a tertiary care hospital.

## MATERIALS AND METHODS

### Bacterial Isolates

A total of 250 nonrepetitive clinical isolates of *K.pneumoniae* were recovered over a period of one year (2014-2015) from our intensive care units (ICUs), i.e. medical ICU (MICU), neurosurgery ICU (NSICU), intensive thoracic unit (ITU), neonatal ICU (NICU), pediatric ICU (PICU), coronary care unit (CCU), and renal ICU (RICU). These isolates obtained from various clinical samples such as endotracheal aspirate (*n* =103), blood (*n* = 56), urine (*n* = 31), pus (*n* = 22), sputum (*n* =3), bronchoalveolar lavage (*n* = 11), central nervous catheter tips (*n* = 13), and sterile body fluids (*n* = 11). The present study was carried out in a tertiary care hospital of Karnataka, South India, with bed strength of 618.

### Antimicrobial Susceptibility Testing

The susceptibilities of the different β-lactam and non-β-lactam antibiotics were tested and the results were interpreted as per the Clinical and Laboratory Standards Institute guidelines^12^. *Escherichia coli* ATCC 25,922 was used as a quality control. The antibiotics were procured from Hi Media, Mumbai, Maharashtra, India.

### Minimum Inhibitory Concentration (MIC)

MIC determination was performed for all the isolates by agar dilution method (CLSI)^12^. Among NDM-1 producers, the MIC of meropenem and colistin ranged between 4-32 mg/mL and 0.25-256 mg/mL, respectively.

### Detection of Extended Spectrum β-lactamase Producers

Isolates resistant or intermediately resistant to aztreonam, cefotaxime and/or ceftazidime were phenotypically detected for the presence of ESBL by the Double Disk Synergy test using cefotaxime (30 µg) and cefotaxime + clavulanic acid (30/10 µg) and (30/10 µg)^12^. *K. pneumoniae* ATCC 700603 was used as the ESBL positive control and *E. coli* ATCC 25,922 was used as the negative control.

### Detection of Carbapenemase Producers

Isolates resistant or intermediately resistant to imipenem, ertapenem and/or meropenem were phenotypically detected for the production of carbapenemases by the modified Hodge test using ertapenem (10 µg) as an indicator disc and by comparing the zone diameter surrounding ertapenem discs supplemented with and without 0.5M EDTA (750 µg), an increase of zone diameter by ≥4 mm suggested the production of metallocarbapenemase^13^.

MICs of meropenem and colistin (Sigma-Aldrich Corporation, St. Louis, US) were determined by the agar dilution method according to the guidelines from the CLSI^12^. The colistin breakpoint was evaluated using breakpoints for *Enterobacteriaceae* recommended by the European Committee on Antibiotic Susceptibility Testing. (Resistant: >2 μg/mL; sensitive: ≤2 μg/mL). *K. pneumoniae* ATCC 700603 was used as a quality control.

### Phenotypic Detection of AmpC Production

AmpC phenotype was detected by means of combined disc method using cefoxitin disc (30 μg) (Hi-media Laboratories, Mumbai), alone and in combination with 400 μg of phenylboronic acid (BA) (Sigma- Aldrich, Fluka, China)^14^.

### DNA Extraction and Amplification

Total DNA was extracted as described by Lee J H^15^. The extracted DNA was subjected to multiplex PCR for the detection of ESBLs and uniplex PCR for NDM and KPC genes^16,17,18^.

The Universal primers used for PCR amplification were as follows:

- *bla*_CTXM_ F-5′-CGCTTTGCGATGCGAG-3′
- *bla*_CTXM_ R-5′-ACCGCGATATCGTTG-3′
- *bla*_TEM_ F-5′-CATTTCCGTGTCGCCCTTATTC-3′
- *bla*_TEM_ R-5′-CGTTCATCCATAGTTGCCTGAC-3′
- *bla*_SHV_ F-5′- GTTCATCCATAGTTGCCTGAC-3′
- *bla*_SHV_ R-5′- AGCCGCTTGAGCAAATTAAAC-3′
- *bla*_NDM_ F-5′- GGTTTGGCGATCTGGTTTTC-3′
- *bla*_NDM_ R-5′- GAATGGCTCATCACGATC-3′
- *bla*_KPC_ F-5′- ATGTCACTGTATCGCCGTCT-3′
- *bla*_KPC_ R-5′- TTTTCAGAGCCTTACTGCCC-3′

## RESULTS

**Graph 1:**
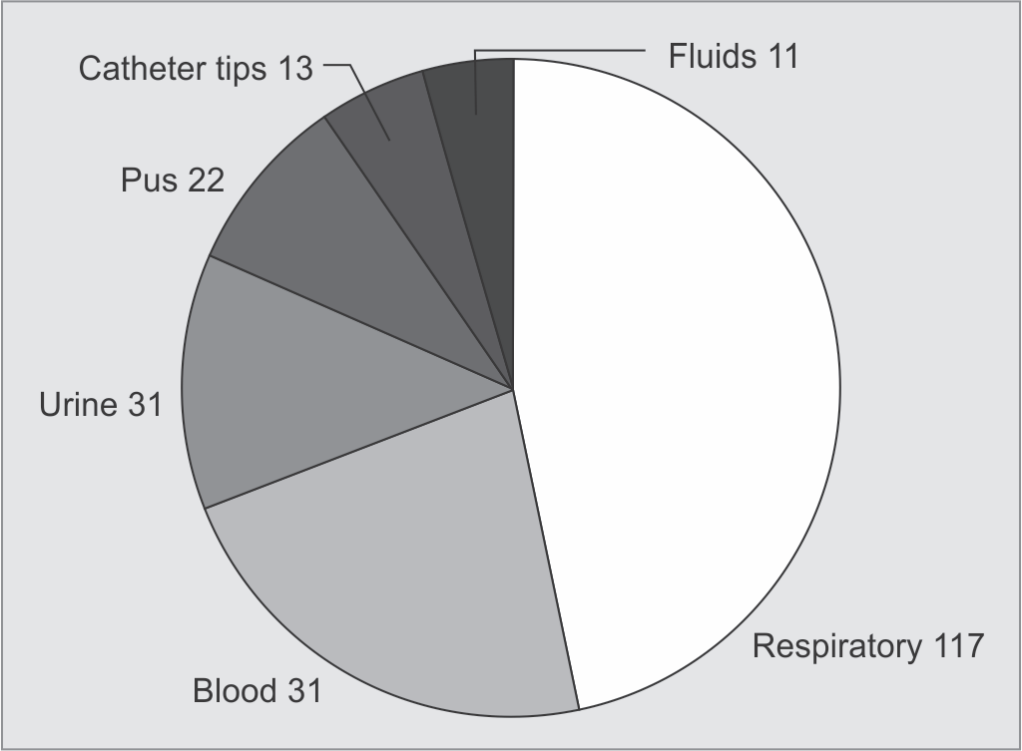
Clinical sources of ESBL producing *K. pneumoniae* isolates

**Graph 2:**
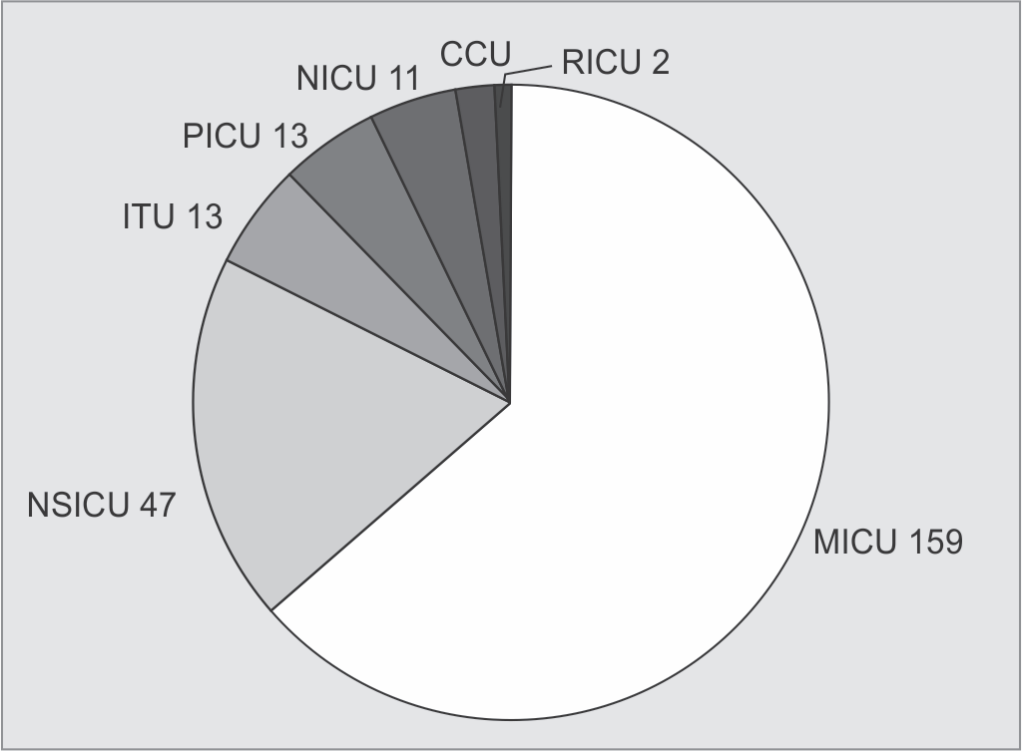
Distribution of *K. pneumoniae* in ICUs

A total of 250 *K. pneumoniae* were isolated from different clinical samples. The distribution of these isolates in clinical specimens and in ICUs is shown in Graphs 1 and 2.

**Graph 3:**
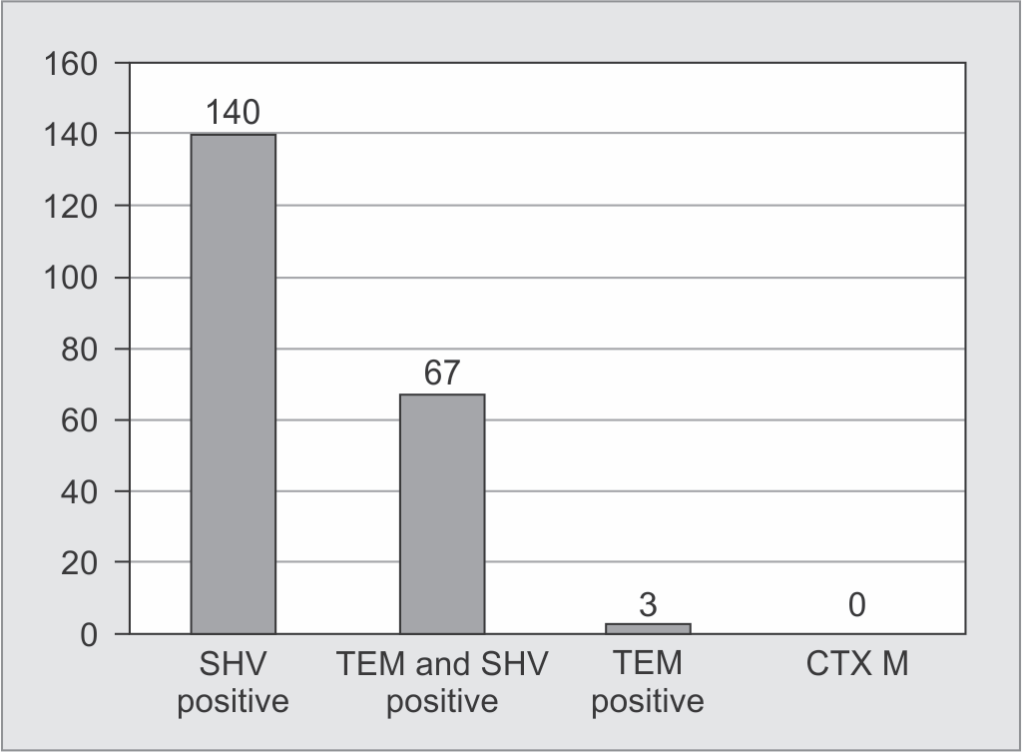
Distribution of *bla* genes among ESBL positive *K. pneumoniae*

**Graph 4:**
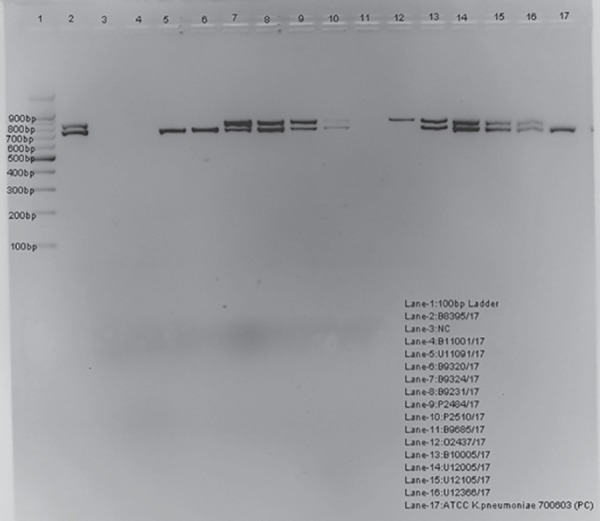
Agarose gel showing multiplex PCR amplified product of *bla*_TEM_ and *bla*_SHV_ genes

Out of 250 *K. pneumoniae* isolates, 210 were screened positive for ESBL producers. The distribution of *bla* genes among ESBL positive *K. pneumoniae* and the gel picture showing the multiplex PCR are given in Graphs 3 and 4.

Lane-1 = 100 bp ladder (Thermo scientific, Made in (EU) Lithuania). Lane −2, 5 and 14 = *bla*_TEM_, *bla*_SHV_ positive amplicons (800 bp and 713 bp, respectively). Lane-3: Negative control (no template DNA added).

The susceptibility of ESBL producers found to be, amikacin (42%), netilimicin (36.5%), imipenem (35%), meropenem (34%), ertapenem (34%), cefaperazone/sulbactum (31%) and piperacillin/tazobactum (28%), respectively.

**Graph 5:**
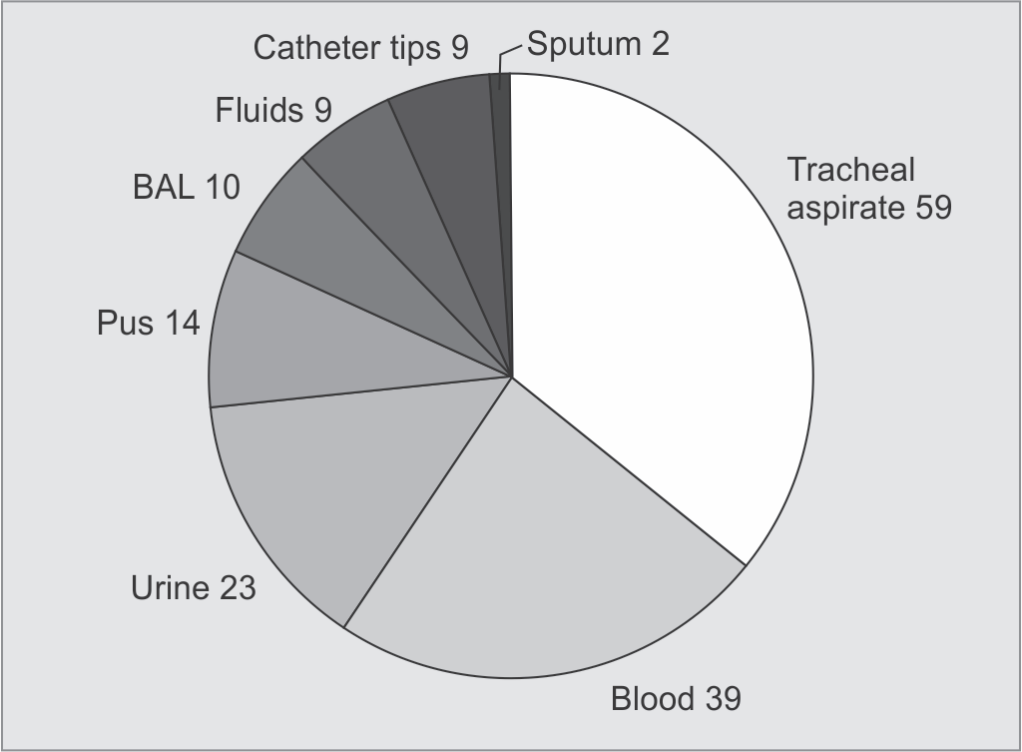
Distribution of carbapenem resistant *K. pneumoniae* in clinical samples

**Graph 6:**
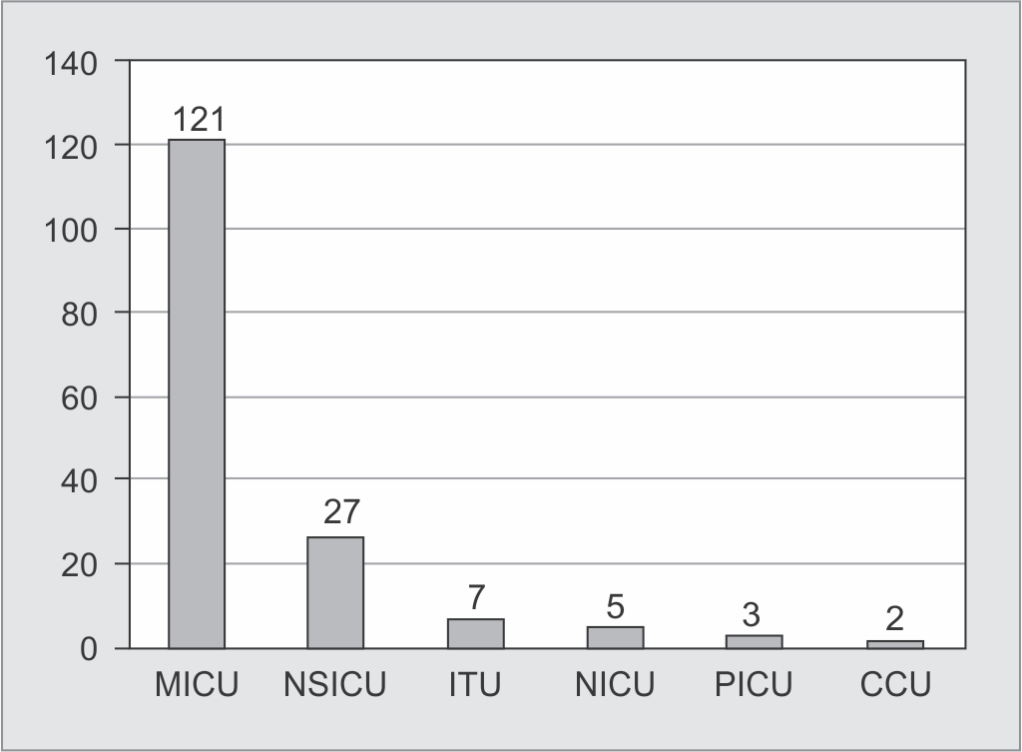
Distribution of carbapenem resistant *Klebsiella pneumoniae* in ICU's

Out of 250 *K. pneumoniae* isolates, a total of 165 (66%) isolates were carbapenem resistant. The distribution of carbapenem resistant *K. pneumoniae* isolates in clinical samples and in ICUs is given in Graphs 5 and 6.

### Prevalence of Carbapenemase Activity Based on Phenotypic tests

The phenotypic detection of carbapenemase production was performed as mentioned in Table 1.

**Table 1:** Carbapenem resistant *Klebsiella pneumoniae* isolates positive for phenotypic tests

*Test*	*Number*	*Percentage (%)*
MHT	96	58.2
EDTA*	28	16.9
AmpC	6	3.6
Total	130	78.7

### Prevalence and Distribution of Carbapenemase Genes

Plasmid DNA was extracted from all the screened positive isolates^15^. The extracted plasmid DNA of each isolate was subjected to PCR detection of the *bla*_NDM-1_ and *bla*_KPC_ genes by using target specific primers^17,18^, which is given in Table 2^17,18^.

**Fig. 1:**
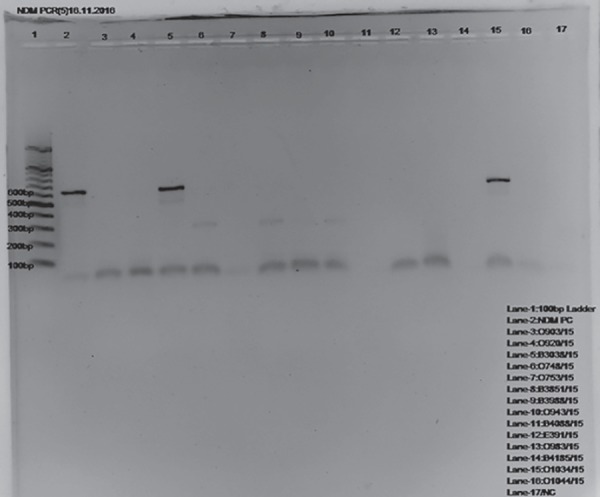
Agarose gel showing PCR amplified product of *bla*_NDM-1_ gene. Lane-1 = 100 bp ladder (Thermo scientific, Made in (EU) Lithuania). Lane −2, 5 and 15= *bla*_NDM-1_ postive amplicons (621 bp). Lane-3: Negative control (no template DNA added)

**Graph 7:**
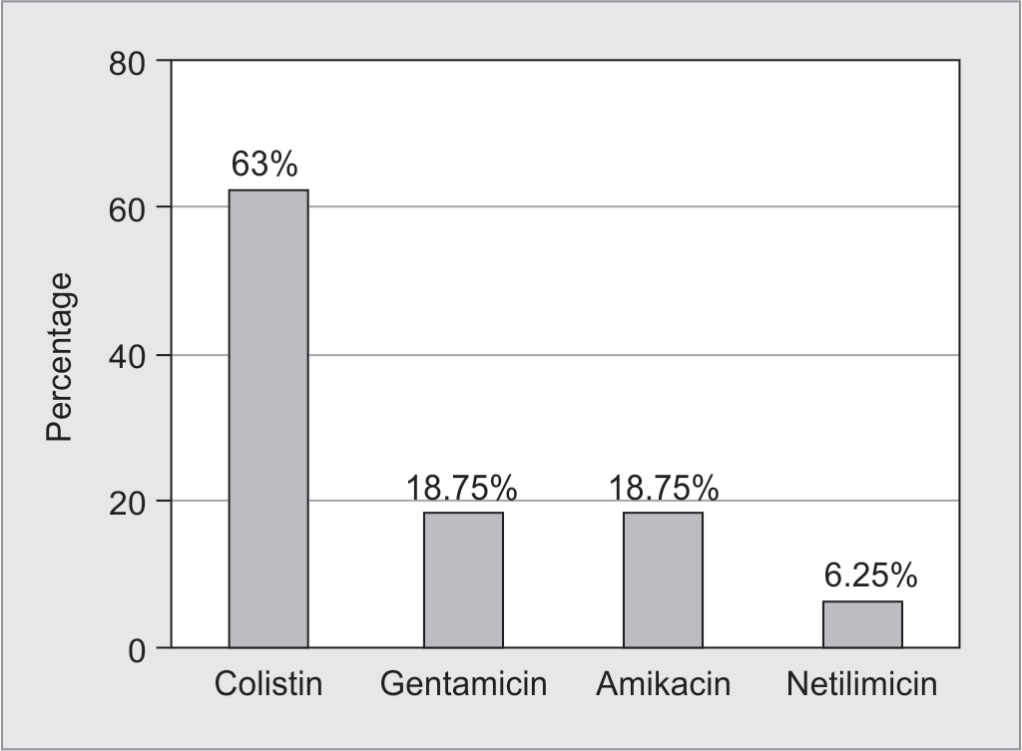
Susceptibility pattern of *bla*_NDM-1_ isolates by disk diffusion

Agarose gel showing PCR amplified product of *bla*_NDM-1_ genes and susceptibility pattern of ***bla***_NDM-1_ isolates is shown in Figure 1 and Graph 7.

### DISCUSSION

*Klebsiella* has been associated with different types of infections and one of the important aspects of *Klebsiella* associated infections is the emergence of multidrug resistant strains particularly those involved in nosocomial diseases.

**Table 2:** Distribution of carbapenemase encoding genes in carbapenem resistant *K. pneumoniae* isolates

*bla Encoding Genes*	*Number*	*Percentage (%)*
*bla*_NDM-1_	16	9.7
*bla*_KPC_	nil	0

In our study, the prevalence of ESBL production was 84% which was higher when compared to other studies. Sharma *et al* found 67.04%, Hoda Hassan *et al* 66.7%, and Mohammed *et al* 30%^19,20,21^. It was similar to a study by Feizabadi *et al*, with 72.1% prevalence^22^. None of the isolates were positive for CTX M gene in our study.

Among these 210 ESBL positive *K. pneumoniae* isolates, 14 (7.8%) of them found to be AmpC producers. A study by Hemalatha *et al* found 7 (9.2%) isolates positive for AmpC^23^. Two other Indian studies reported 8% and 43% AmpC producers^24,25^.

*K. pneumoniae* have acquired carbapenemases, the enzymes capable of breaking down most β-lactams including carbapenems, and confer resistance to these drugs. Reports indicate that carbapenemase producing enterobacteriaceae isolates seem to be increasing in number in the last few years. In our study, we found 66% of the isolates were carbapenemase producers. Gupta *et al*., from All India Institute of Medical Sciences, New Delhi in 2006 found that resistance to meropenem was 6.9%^26^. Nagaraj *et al* observed 75% of the *K. pneumoniae* isolates were carbapenem resistant in their study^8^. Aseem *et al* stated that 35.3% of the isolates in their study were resistant to carbapenems^27^.

Genotypic analysis of these carbapenem resistant isolates revealed the prevalence of the *bla*_NDM-1_ as 9.7% and none of the isolates were positive for *bla*_KPC_ in our study. The epidemiology of *K. pneumoniae* producing KPCs varies geographically. Globally, the highest rate of carbapenem resistance has been reported in Greece with 68% resistance (KPC, OXA-48-like, NDM) followed by India (NDM, OXA-48-like, KPC) and eastern Mediterranean regions (NDM, OXA-48-like) with 54% resistance. USA, China (KPC, NDM, OXA −48-like) and Africa (OXA-48-like, NDM) have low resistance rates with 11, 11 and 4% respectively^28^.

The predominant enzymatic mechanism of resistance in Europe is KPC followed by OXA-48-like and NDM, while in USA; it is KPC followed by NDM and minimal due to OXA-48-like^29^. Several studies in Spain showed that most carbapenemase-producing *K. pneumoniae* harbored OXA-48-like or class B carbapenemases, and that of KPC-producing *K. pneumoniae* was very low (2-3%)^30,31^.

Lascos *et al*. reported 34.8% prevalence of *bla*_KPC_ in CRE and Aseem *etal*. observed only 3.7% of*bla*_KPC_ out of 35.2% carbapenem resistant *Klebsiella pneumoniae* isolates^8,30^.

However, in our study, none of the isolates were positive for *bla*_KPC_. This correlates with findings of Nagaraj *et al*. who has observed 75% of *bla*_NDM_ but not detected*bla*_KPC_ from any of the carbapenem resistant isolates^8^. A study from Vellore, by Veeraraghavan *etal* showed the coexpression of *bla*_NDM_ and *bla*_OXA48_ in 28%, *bla*_NDM_ 19%, *bla*_OXA48_ in 13% and *bla*_KPC_ was absent among the carbapenem resistant isolates^33^. The endemic spread of NDM-producing *K. pneumoniae* has also been reported in the UK, which has close relationships with India and Pakistan^32^.

The Balkan states, the Arabian Peninsula, and North African countries have also been recently considered as an additional reservoir of NDM producers^32–35^. In the Arabian Peninsula, NDM-1 was the most frequently encountered carbapenemase 46.5%, followed by OXA-48-like carbapenemases 32.5%^36^. In India, a study by Deshpande *et al*, reported NDM-1 was the most common carbapenemase type detected and accounted for 75.2% of the carbapenemase-producing isolates^37^. In another study, an incidence of *bla*_NDM-1_ in a single*K.pneumoniae* isolate was reported from a surgical site infection^38^. In our study we found 9.7% of NDM-1 which is similar to a study from tertiary care hospital of Northeast India where they found 8.67% of NDM pocessing *Klebsiella pneumoniae* isolates^39^.

It was observed that the coexistence of *bla* gene along with NDM producers found to be 69% and 50% of SHV and TEM respectively. Previous studies from India have reported the coexistence of *bla* gene along with NDM producers^39–41^. Similar studies from abroad also showed the presence of *bla* genes along with NDM producers^42,43^ .

We found 78.7% of the CRKP were positive for the production of one or more carbapenemase mechanisms phenotypically. It has been observed that among the screened CRKP, 9.7% isolates found to harbor *bla*_NDM-1_ gene. Rest of the CRKP isolates did not possess any of the resistant mechanisms tested. The important contributing factors leading to carbapenem resistance in these isolates might be due to the hyper production of ESBL, or other enzymatic mechanisms of carbapenem resistance like, IMP, VIM, and OXA, porin loss or efflux pumps^44,45^.

## CONCLUSION

In conclusion, carbapenem resistant *K. pneumoniae* have been considered as one of the greatest threats to the global health care in this century. The prevalence of KPC gene i.e., *bla*_KPC_ in carbapenem resistant isolates from our geographical area (South Western India) seems to be very less. Pathogens that produce carbapenemases along with an ESBL or AmpC β-lactamases are particularly challenging for clinicians and are a major threat worldwide. Moreover, the widespread dissemination of the new NDM-1 metallo-β-lactamase requires particular attention as the genetic background demonstrates extreme mobility and versatility. The dissemination of such plasmids between different clinically important bacterial species may lead to serious public health issues, as *K. pneumoniae* accounts for one of the important bacterial species in the dissemination of antibiotic resistance genes; particularly in hospital environments. Therefore, the early detection of the *bla*_NDM-1_ possessing *K. pneumoniae* isolates is necessary with any reduced susceptibility to the carbapenems. Timely intervention in the form of rapid detection, good infection control practices and judicious use of antibiotics will ensure that the spread of drug resistance among bacteria can be kept under control.
